# Bioremediation of Petroleum Hydrocarbons in Seawater: Prospects of Using Lyophilized Native Hydrocarbon-Degrading Bacteria

**DOI:** 10.3390/microorganisms9112285

**Published:** 2021-11-03

**Authors:** Rafaela Perdigão, C. Marisa R. Almeida, Catarina Magalhães, Sandra Ramos, Ana L. Carolas, Bruno S. Ferreira, Maria F. Carvalho, Ana P. Mucha

**Affiliations:** 1 CIIMAR-Interdisciplinary Centre of Marine and Environmental Research, University of Porto, Terminal de Cruzeiros do Porto de Leixões, Av. General Norton de Matos, S/N, 4450-208 Matosinhos, Portugal; calmeida@ciimar.up.pt (C.M.R.A.); cmagalhaes@ciimar.up.pt (C.M.); ssramos@ciimar.up.pt (S.R.); mcarvalho@ciimar.up.pt (M.F.C.); amucha@ciimar.up.pt (A.P.M.); 2 School of Medicine and Biomedical Sciences (ICBAS), University of Porto, Rua de Jorge Viterbo Ferreira, 228, 4050-313 Porto, Portugal; 3 Faculty of Sciences, University of Porto (FCUP), Rua do Campo Alegre 790, 4150-171 Porto, Portugal; 4 Biotrend S.A., Biocant Park, Núcleo 04 Lote 2, 3060-197 Cantanhede, Portugal; alcarolas@biotrend.pt (A.L.C.); bsferreira@biotrend.pt (B.S.F.)

**Keywords:** autochthonous bioremediation, oil spills, hydrocarbons, bioaugmentation, bioremediation agent, lyophilized bacteria, biotechnology, marine environment

## Abstract

This work aimed to develop a bioremediation product of lyophilized native bacteria to respond to marine oil spills. Three oil-degrading bacterial strains (two strains of *Rhodococcus erythropolis* and one *Pseudomonas* sp.), isolated from the NW Portuguese coast, were selected for lyophilization after biomass growth optimization (tested with alternative carbon sources). Results indicated that the bacterial strains remained viable after the lyophilization process, without losing their biodegradation potential. The biomass/petroleum ratio was optimized, and the bioremediation efficiency of the lyophilized bacterial consortium was tested in microcosms with natural seawater and petroleum. An acceleration of the natural oil degradation process was observed, with an increased abundance of oil-degraders after 24 h, an emulsion of the oil/water layer after 7 days, and an increased removal of total petroleum hydrocarbons (47%) after 15 days. This study provides an insight into the formulation and optimization of lyophilized bacterial agents for application in autochthonous oil bioremediation.

## 1. Introduction

Hydrocarbon pollution resulting from anthropogenic activities threatens our marine ecosystems, whether by acute events of contamination, such as oil spills, or by chronic contamination. Mass-scale oil spills have high media coverage and are extremely dangerous to the environment, pressuring governments and agencies to act fast to contain and tackle the spillage. Sometimes, a faster response might not be the most environmental-friendly or effective approach [1], as illustrated by the Deepwater Horizon oil spill in 2010, where chemical dispersants were applied on a large scale to disperse the oil, harming the wildlife [2,3]. Bioremediation technologies, on the other hand, have been considered promising ecologic alternatives to tackle oil spills, avoiding the negative implications associated with physical-chemical techniques, like the introduction of chemical dispersants or burning the spilled oil [4,5,6]. Bioremediation can be divided into two strategies: biostimulation (BS), by adding nutrients to the affected area to stimulate the oil-degrading metabolisms by the microbial community naturally present, and bioaugmentation (BA) where known oil-degrading microorganisms are added to increase their abundance among the local microbial community. A combination of these two methods has been proved to enhance hydrocarbon degradation [4,7,8]. Many groups of microorganisms have been reported to play a role in hydrocarbons degradation in the marine environment, such as the filamentous fungi genera *Aspergillus* and *Penicillium* [9] and the yeast genus *Candida* [10], nonetheless, bacteria are considered the major intervenient in hydrocarbons biodegradation at sea [11]. Members of the classes *Gammaproteobacteria*, *Alphaproteobacteria* and *Actinobacteria* have been isolated from seawater and marine sediment samples showing hydrocarbon bioremediation potential [12,13,14]. When considering the application of bioaugmentation techniques to tackle oil spills, the use of a consortium of microorganisms may be more effective than using single bacterial strains, since different groups of bacteria can metabolize different groups of hydrocarbons [15,16], by producing different oil-degrading enzymes [17] and biosurfactants [18]. Previous studies [5,19,20] observed a higher hydrocarbon-degrading efficiency when using a consortium of microorganisms in seawater. Furthermore, the use of autochthonous microorganisms can improve the degradation efficiency, considering that these organisms are better adapted to the environment. These microorganisms have the advantage of not competing with the natural community for the carbon source and do not cause the possible negative impact associated with the introduction of exogenous organisms [21,22]. Recently, several laboratory studies highlighted the importance and potential of using native bacterial consortia to bioremediate petroleum hydrocarbons in impacted marine environments [5,19,20].

Even by doing effective laboratory experiments to test bioremediation agents or a combination of agents, the bioremediation efficiency in a real environment relies on various factors, such as environmental conditions, the concentration and chemical composition of the oil spilled, the bioavailability of hydrocarbons, the concentration of nutrients present and the time taken to develop a tailor-made solution and effective bacterial consortium to apply after the oil spill [23,24,25].

Regarding the application of bioremediation agents in open water systems, such as seawater, the application of free-cell bioremediation might be challenging as it might disperse in the water column [26]. To tackle this issue, some studies suggest the application of immobilized bacterial cells into carriers, biofilms or enzyme substances to enhance the hydrocarbons biodegradation performance [27,28,29,30,31]. Wang et al. [28] studied the application of immobilized bacteria in field tests (3 months), contaminated with crude oil and diesel and observed that the immobilized bacterial consortia performed better in the degradation of hydrocarbons. Hou et al. [31] observed an enhanced diesel bioremediation process by immobilizing an oil-degrading bacteria *Acinetobacter* sp. to a novel carrier when compared to an addition of free cells to the experiment. Despite the promising bioremediation results from these previous studies, most of the immobilized bacteria used in the process were not autochthonous for the site.

Field tests with the application of microbial agents in the marine environment evidenced the effectiveness of bioaugmentation in the removal of oil from rocks and sediments at a beach impacted by the Nakhodka oil spill (1997) [32]. There are already a few microbial products in the market to tackle oil spills, besides nutrient fertilizers and other chemical compounds prepared for biostimulation. A market study on patents of microorganisms to be applied in bioremediation carried out by Villela et al. [33], revealed that most of these patents belong to microorganisms from the Bacteria group (368 out of 500 patents). Species from the genera *Pseudomonas*, *Rhodococcus*, *Acinetobacter* and *Bacillus* are in the top 10 of bacterial bioremediation agents. However, most patents considered only individual bacterial strains or consortia of exogenous microorganisms. Regarding the use of autochthonous bacterial consortia to bioremediate petroleum hydrocarbons in seawater only one patent was found [34]. Thus, more research is needed on the identification/isolation of autochthonous bacterial strains with high petroleum biodegradation potential to increase the range of native bacterial consortia products for hydrocarbons bioremediation applications. Moreover, the procedures needed to fully use those bacterial strains after isolation, either alone or in a consortium, must be studied. This includes the optimization of biomass production, biomass preparation for field application and the amount of biomass needed to be introduced considering the size of the spill. To fulfil this need, and facilitate the preservation of the bacterial biomass, a lyophilization (or freeze-drying) technique can be used [35]. By doing so, the resulting bacterial product, in the form of powder, could be more easily stored and transported, at temperatures between 2 °C and 8 °C, occupying less volume as opposed to in solution, and allow the direct application of the bacterial product to an oil spill. This process could also increase the “shelf-life” of the bacterial product, as bacterial cells can retain their viability after 5–35 or even 50 years from the lyophilization process [36].

Taking, as a case study, a beach located near an oil refinery, the present work aimed to (i) optimize the biomass production of three oil-degrading bacterial strains previously isolated from beach seawater and sediment, for lyophilization purposes; (ii) test the viability and hydrocarbon-degrading capacity of the lyophilized strains, individually or combined into a consortium; (iii) optimize the ratio of the bacterial consortium biomass and oil for efficient biodegradation of hydrocarbons and (iv) validate the bioremediation efficiency of the optimized lyophilized consortium in microcosms experiments with natural seawater from the case study beach.

## 2. Materials and Methods

### 2.1. Growth Optimization of Hydrocarbon-Degrading Bacteria for Lyophilization

Three bacterial strains, *Rhodococcus erythropolis* CPN 2, *R. erythropolis* CPN 3 and *Pseudomonas* sp. 1.7 L, previously isolated from water and sediment collected in the beach Cabo do Mundo (41°13′13.9′′ N 8°42′53.1′′ W), NW Portugal [37], were used in the current study due to their high potential to degrade petroleum hydrocarbons. This beach is located near an oil refinery and about 4 km from the Leixões Harbour (Matosinhos, Portugal). After isolation, the bacterial strains were cryopreserved at −80 °C [8]. For the current study, laboratory growth optimization tests were initially conducted for each bacterial strain using different carbon sources alternative to hydrocarbons, namely sodium acetate, glycerol, glucose and peptone. These experiments were carried out in batch mode, in 250 mL Erlenmeyer flasks containing 50 mL of Bushnell–Haas (BH) medium (Difco) supplemented with 2% NaCl (*v*/*v*) and 10 g L^−1^ of one of the indicated carbon sources. A bacterial inoculum was prepared for each cryopreserved bacterial strain, and individually used to inoculate the flasks at an initial biomass density of ca. 0.05 (optical density (OD), measured by spectrometry at 600 nm). After selecting a suitable carbon source for biomass production of each bacterial strain (Figure S1), the bacterial strains were lyophilized. For the lyophilization process, each cryopreserved bacterial strain was first grown in 25 mL of Nutrient Broth (NB, Merck cat number 1054430500, 13 g L^−1^), a medium that contains peptone, supplemented with 10 g L^−1^ of sodium acetate, at an initial OD (at 600 nm) of ca. 0.1. All cultures were incubated in an orbital shaker (150 rpm) at 30 °C. After this period, the cultures were centrifuged (10 min, 9000 rpm, 25 °C) and the resulting pellet was resuspended in 30 mL of cryopreservation medium CP01 (Nutrient broth 13 g L^−1^ and Sucrose 100 g L^−1^). Tubes were left at room temperature for 1 h and then stored at −80 °C. Afterward, the cells were lyophilized for 63.5 h (−50 °C, 0.3 bar), after which were stored in closed recipients and preserved at 5 °C. The cell viability and the hydrocarbon degradation potential, estimated by the most probable number (MPN) method, were assessed in laboratory experiments using the lyophilized bacterial strains alone or in a consortium (Section 2.2.1).

### 2.2. Evaluation of the Viability and Hydrocarbon-Degradation Potential of the Lyophilized Bacteria

#### 2.2.1. Experiments with the Lyophilized Strains CPN2, CPN3, 1.7 L

The viability and degradation potential of each lyophilized strain, alone or in a consortium, were tested in 100 mL serum flasks containing different media artificially contaminated with sterile petroleum (supplementary material Figure S2). The following treatments were applied: (i) sterile seawater, petroleum and lyophilized inoculum (SPI), (ii) sterile seawater, petroleum, lyophilized inoculum and the addition of nutrients (KNO_3_ (40 mM) and KH_2_PO_4_ (8 mM)) (SPNI), (iii) nutrient broth, petroleum and lyophilized inoculum (NBPI). Two controls were also assembled, one with sterile seawater and petroleum and the other with sterile seawater, petroleum and nutrients (SPN). Each treatment was tested in triplicate. For the SPI, SPNI and NBPI treatments, initial suspensions of the lyophilized strains CPN 2 (2i), CPN 3 (3i) and 1.7 L (4i) were prepared in 250 mL flasks, with a medium/bacterial lyophilizate ratio of 30:0.05 (*v*/*w*), where 0.05 g of lyophilized bacteria and a final volume of 30 mL of seawater were applied. The consortium (5i) was prepared in 250 mL flasks containing 90 mL of the respective media and 0.05 g of each lyophilized strain. After an hour of hydration, 10 mL of the suspension was transferred to 100 mL serum flasks and the nutrients were added to the SPN and SPNI treatments. In parallel, each lyophilized strain or the consortium was suspended, according to the ratio indicated above, in Bushnell-Haas (BH) medium supplemented with 2% NaCl (*v*/*v*), and immediately (with no hydration period) evaluated for oil degraders abundance by the MPN method. Each treatment was tested in triplicate and with the addition of 0.25 mL of petroleum (0.2 µm filtered). All flasks were closed with sealing caps to maintain the sterility of the medium and evaluate the hydrocarbon-degrading potential of only the lyophilized bacteria added to the media. The flasks were incubated for 24 h under constant agitation (100 rpm), in the dark and at room temperature (ca. 25 °C). The abundance of hydrocarbon-degraders was evaluated for each condition, after one hour (T0) and 24 h (T1) of incubation with petroleum, by the MPN method.

#### 2.2.2. Experiments with Different Concentrations of the Lyophilized Consortium

In this experiment, the consortium of the lyophilized strains was tested at different concentrations, in 100 mL serum flasks containing natural seawater collected at the Matosinhos beach (salinity 36, pH 8) (41°10′35.033″ N 8°41′33.605″ W). An initial suspension was created consisting of a mixture of 0.05 g of each lyophilized strain hydrated in 90 mL of natural seawater (6i) for one hour, corresponding to an initial concentration of 1.7 g L^−1^ for each lyophilized strain. Afterward, two successive tenfold dilutions were prepared in the natural seawater (7i and 8i), corresponding to final concentrations of 1.7 × 10^−1^ g L^−1^ and 1.7 × 10^−2^ g L^−1^ of each lyophilized strain. The experiment set up is summarized in Figure S3. The treatments SPI, SPNI and the controls SP and SPN were assembled with natural seawater, in triplicate, with the addition of 0.25 mL of petroleum (0.2 µm filtered). All flasks were closed and incubated for 24 h, under constant agitation (100 rpm), in the dark and at room temperature (ca. 25 °C). The MPN was analyzed in the natural seawater (Ti), after one hour (T0) and 24 h (T1) of incubation with petroleum.

### 2.3. Microcosms Bioremediation Experiment in Natural Seawater

To evaluate the biodegradation efficiency of the optimum concentration of the lyophilized consortium in natural seawater, a microcosms experiment was assembled. The experiment was carried out in 100 mL serum flasks containing 10 mL of natural seawater collected from a beach in Matosinhos (41°10′35.033″ N 8°41′33.605″ W), and petroleum (0.2 µm filtered) in the ratio of 20:0.5 (*v*/*v*). Three different treatments were tested: (i) natural attenuation (NA) (seawater + petroleum), (ii) biostimulation (BS) (seawater + petroleum + nutrients), and (iii) a combination of biostimulation and bioaugmentation (BA) (seawater + petroleum + nutrients + lyophilized consortium). The overall scheme is represented in Figure S4. Each treatment was tested in triplicate except for NA, for which 6 additional flasks were prepared at the beginning of the experiment and preserved at −20 °C for analysis of total petroleum hydrocarbons (TPHs) at the initial time. Considering the results obtained in the experiments of Section 2.2.2, the consortium concentration in the order of 10^−1^ g L^−1^ was selected as the inoculum for the BA treatment of the microcosms experiment. All flasks were closed and incubated for 15 days, under constant agitation (100 rpm), in the dark and at room temperature. The abundance of hydrocarbon-degrading bacteria in solution (2 mL collected from each flask) was analyzed for the initial natural seawater and after one hour (T0), 24 h (T1), 7 days (T7) and 15 days (T15) of incubation with petroleum. Aliquots of initial seawater and of each solution were also collected for isolation of potential oil-degrading bacterial strains, for the treatment BA at T0, T7 and T15 and for the treatments BA and BS at T15. At the end of the 15 days, the remaining solution of each treatment was stored at −20 °C in the respective flask for TPHs analysis.

### 2.4. Abundance of Hydrocarbon-Degrading Bacteria by the MPN Method

To assess the abundance of hydrocarbon-degrading bacteria, the most probable number (MPN) method adapted from [38] as reported in Almeida et al. [4] was performed. In 96-well plates, 20 μL of the sample was inoculated into 180 μL sterile BH medium supplemented with 2% NaCl together with 10 μL of petroleum (0.2 µm filtered), in tenfold dilutions. After two weeks of incubation at room temperature (ca. 25 °C), 50 μL of sterilized iodonitrotetrazolium solution (3 g L^−1^) was added to each well. After an overnight incubation, positive wells (with the color violet) were registered.

### 2.5. Analyses of the Total Petroleum Hydrocarbons (TPHs)

The flasks with the remaining solution from the microcosm experiment were first defrosted. Then, 20 mL of tetrachloroethylene was added to each flask, and the flask was agitated to detach the petroleum from the walls and subjected to an ultrasonic bath for 15 min for hydrocarbons extraction, as described in Almeida et al. [4]. TPHs were analyzed in the extract by Fourier transform infrared spectrophotometry (Jasco FT/IR-460 Plus) as described in Almeida et al. [4]. The evaluation of TPHs was chosen as this methodology mostly quantifies saturated hydrocarbons and these types of hydrocarbons are normally the first ones to be degraded (e.g., [39]).

### 2.6. Isolation of Potential Hydrocarbon-Degrading Bacteria

A composed sample resulting from the combination, for each treatment, of aliquots collected from the triplicate flasks, was prepared, being afterward, ten-fold diluted in sterile saline solution (0.85%) and spread onto M1 agar medium (1 L of seawater, 10 g soluble starch, 4 g yeast extract, 2 g peptone and 15 g agar) plates and incubated at 28 °C, for 3 days. Morphologically different colonies were described and isolated by the streaking method in M1 agar. Pure colonies were preserved in 21% glycerol at −80 °C and biomass of each bacterial strain was collected for DNA extraction.

### 2.7. Identification of Bacterial Strains

The DNA of the bacterial strains isolated during the microcosm bioremediation experiment (Section 2.6) was extracted by using the commercial kit E.Z.N.A.^®^ Bacterial DNA Kit (Omega, bio-tek), following the protocol provided by the supplier. For phylogenetic identification, the regions V1 to V9 of the 16S rRNA gene were amplified using the universal primers 27F (5′ AGAGTTTGATCMTGGCTCAG 3′) and 1492R (5′ TACGGYTACCTTGTTACGACTT 3′). The PCR reaction mixture, with a final volume of 10 μL, contained: 5 μL of Qiagen Multiplex PCR Master Mix (Qiagen, CA, USA), 1 μL of each primer (2 mM) and 3 μL of DNA sample. PCR conditions were as follow: a first cycle of 15 min at 95 °C; followed by 30 cycles at 94 °C for 30 s, 48 °C for 90 s and 72 °C for 2 min; a final cycle at 72 °C for 10 min. The amplified DNA samples were visualized in a 1.5% agarose gel containing SYBR Safe (Thermo Fisher Scientific, Waltham, MA, USA). PCR products were sequenced at Genomics i3S Scientific Platform (Porto, Portugal). The resulting forward and reverse 16S rRNA sequences were aligned using the Geneious software (version 11.1.4), and the consensus sequences were compared to those present in the nucleotide collection database of the National Center for Biotechnology Information (NCBI, Bethesda, MD, USA) and two other databases, to confirm the results, EZTaxon database (http://www.ezbiocloud.net, accessed on 18 January 2021) and Ribosomal Database Project (https://rdp.cme.msu.edu/index.jsp, accessed on 18 January 2021). The 16S rRNA gene sequences of the identified strains were deposited in GenBank (NCBI) under the accession numbers indicated in Table S1.

To construct a phylogenetic tree, an alignment was first made with the sequences of all isolated bacterial strains and their three closest neighbor sequences in Genbank, using the MUSCLE alignment tool from the Geneious software. Then, a maximum likelihood phylogenetic tree was generated with 1000 bootstraps based on the Tamura-Nei model using the MEGA X program (Version 7.0, PA, USA) [40].

### 2.8. Data Analysis

Triplicates of MPN concentrations from the experiments with lyophilized strains and microcosm experiments were analyzed and their mean values (*n* = 3) and standard deviations calculated. The same approach was applied to the determination of the TPHs concentration in the microcosm bioremediation experiments. For both MPN and TPHs, statistical analyses were made with IBM SPSS statistics program (version 26, IBM, Armonk, NY, USA), where a non-parametric Kruskal–Wallis ANOVA multiple comparison test was applied. Significant differences were considered when p values were equal or below 0.05.

## 3. Results

### 3.1. Abundance of Hydrocarbon-Degraders in the Experiments with Lyophilized Strains

Laboratory growth optimization tests were initially conducted for each bacterial strain using carbon sources alternative to hydrocarbons, namely sodium acetate, glycerol, glucose and peptone. Results indicated that sodium acetate, glucose and peptone were good alternative carbon sources for biomass growth of the CPN2 and CPN3 strains, whilst glucose and peptone promoted the biomass growth for the 1.7 L strain (Figure S1). After obtaining this information, the bacterial strains were lyophilized as described in materials and methods (Section 2.1), to obtain a bioremediation product.

#### 3.1.1. Experiments with the Strains CPN2, CPN3, 1.7 L and Their Consortium

**Figure 1:**
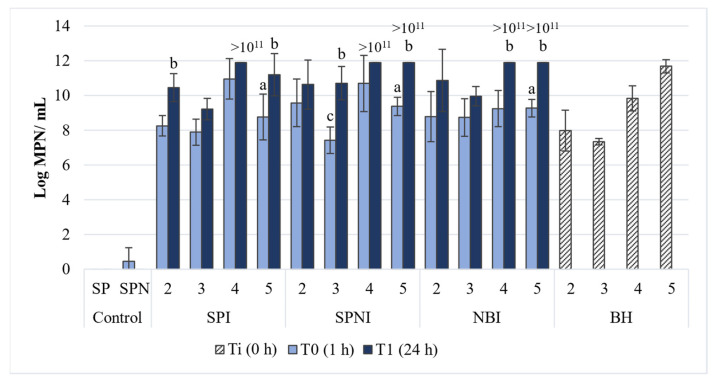
Abundance of hydrocarbon degraders, evaluated by the most-probable number (MPN) method, for the lyophilized bacterial strains CPN2 (2), CPN 3 (3), 1.7 L (4), and their consortium (5) (mean value, standard deviation, *n* = 3) after direct application in BH medium (BHI), after 1 h (T0) and after 24 h (T1) of incubation in the treatments SPI (sterile seawater + petroleum + inoculum), SPNI (sterile seawater + petroleum + nutrients + inoculum), NBPI (nutrient broth + petroleum + inoculum). a—significant differences comparing all treatments with the values from BHI treatment, for each lyophilized bacterium (or their consortium); b—significant differences comparing with the values from T0, within the same treatment and type of lyophilized bacteria; c—significant differences comparing different type of lyophilized bacteria, in the same treatment at T0.

After the lyophilization process, the viability and capacity for hydrocarbon degradation of the three lyophilized bacterial strains (CPN2, CPN3 and 1.7 L), either individually or as a consortium, was evaluated in sterile seawater. For the treatments inoculated with the lyophilized bacterial strains (SPI, SPNI and NBPI), high abundance of hydrocarbon-degraders was observed after just 1 h of incubation with petroleum (between 10^7^ and 10^11^ MPN/mL), having all strains increased after 24 h, reaching values between 10^9^ and >10^11^ MPN/mL (above the operational limit of the method). These high abundances were also observed when the lyophilized bacteria were immediately dissolved in BH medium, without the need of contact with petroleum. All this indicates that the lyophilized bacterial strains maintained their natural capacity to degrade petroleum hydrocarbons and that they can proliferate if this carbon source is available. In the control treatments, consisting in sterile seawater doped with petroleum and with (SPN) or without nutrients, the abundance of hydrocarbon degraders after 1 h and 24 h of incubation was approximately zero, confirming the sterility of the medium in this assay (Figure 1). Overall, the lyophilized strains presented a similar abundance of hydrocarbon degraders, for each time, across all treatments, with no statistical differences between treatments with the same lyophilized bacterial, both at T0 and T1. Within the SPI treatment, at T0, the addition of the 1.7 L lyophilized strain, resulted in a significantly higher MPN value, when compared to the other strains. Since the consortium of the three strains presented densities higher than 10^11^ MPN/mL and given the advantage of using diverse strains in the biodegradation of petroleum hydrocarbons, the consortium was selected for further experiments.

#### 3.1.2. Experiments with Different Concentrations of the Consortium of the Three Lyophilized Strains

**Figure 2:**
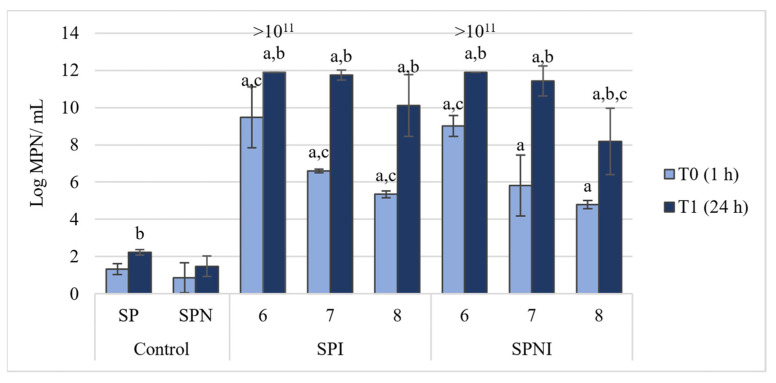
Abundance of hydrocarbon-degraders, evaluated by the most-probable number (MPN) method (mean value, standard deviation, *n* = 3), in controls SP (natural seawater + petroleum) and SPN (natural seawater + petroleum + nutrients), and in treatments SPI (natural seawater + petroleum + inoculum) and SPNI (natural seawater + petroleum + nutrients + inoculum), where a consortium of three lyophilized strains (inoculum) was added to SPI and SPNI in different concentrations: 1.7 (6), 1.7 × 10^−1^ (7), 1.7 × 10^−2^ (8) g L^−1^. a—significant differences comparing all treatments with the respective control, at the same time; b—significant differences comparing with the values at T0, in the same treatment and concentration; c—significant differences comparing with the values from other concentrations within the same treatment, at the same time.

The hydrocarbon-degrading potential of the consortium containing the three lyophilized bacterial strains (CPN2, CPN3 and 1.7 L) at different concentrations (in the order of 1.7 g L^−1^, 10^−1^ g L^−1^and 10^−2^ g L^−1^) was tested in natural seawater after incubation for 1 h and 24 h with petroleum (Figure 2). The control treatments (SP and SPN) had low abundance of hydrocarbon degraders for the same period of incubation, around 10^1^ MPN/mL, with no significant differences between T0 and T1. All treatments and consortium concentrations had significantly higher MPN values than the respective controls at T0 and T1. In both SPI and SPNI treatments, the lower the concentration of lyophilized consortium, the lower the MPN values at T0. After 24 h (T1), the higher concentration of the lyophilized consortium (in the order of 1.7 g L^−1^) presented an abundance of hydrocarbon degraders higher than the operational limit of the method (>10^11^ MPN/mL), but this value was not significantly different from the other lyophilized concentrations in the SPI treatment. With the addition of nutrients, in the SPNI treatment, the two higher concentrations of the lyophilized consortium (in the order of 1.7 g L^−1^ and 10^−1^ g L^−1^) displayed the highest performance, with values of 10^11^ MPN/mL or higher. No significant differences were observed, comparing MPN values of each consortium concentration, between SPI and SPNI treatments, in both T0 and T1.

Based on the results of this assay, the consortium with intermediate concentration (in the order of 10^−1^ g L^−1^) was selected for the microcosms experiment since it displayed a potential similar to the consortium 10 times more concentrated to biodegrade petroleum hydrocarbons.

### 3.2. Microcosm Bioremediation Experiment

To evaluate the biodegradation efficiency of the optimized consortium (Section 3.1.2) in natural seawater, a microcosm experiment was assembled, with three treatments: natural attenuation (NA), biostimulation (BS) and bioaugmentation (BA). The hydrocarbon biodegradation efficiency was tested after 1 h, 24 h, 7d and 15 days of incubation with petroleum.

**Figure 3:**
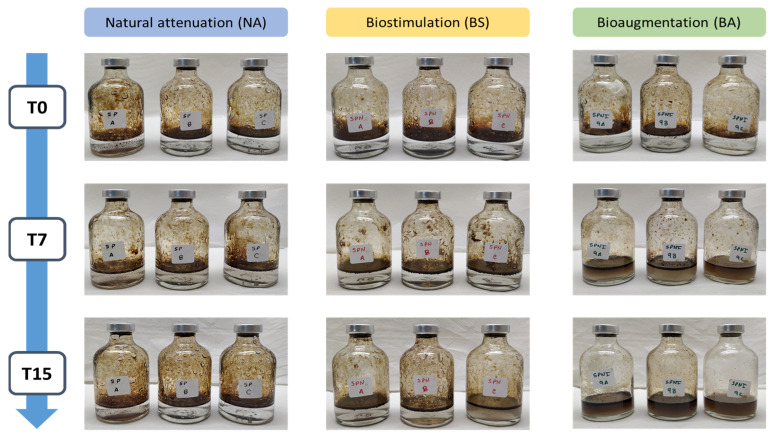
Visual aspect of the microcosm flasks at the beginning (T0), after 7 days of experiment (T7) and at the end of the experiment (after 15 days) (T15), for the different treatments: natural attenuation (NA), biostimulation (BS) and bioaugmentation with an inoculum of lyophilized consortia.

For each treatment, photos were taken at the beginning and after 7 and 15 days of experiment (Figure 3). At the beginning of the experiment (T0), a clear separation between the oil slick and the medium was observed for the 3 treatments. After 7 days of experiment (T7), this separation was still observed for natural attenuation (NA) and biostimulation (BS) treatments, while for bioaugmentation (BA) treatment a clear blending between the petroleum and the medium was observed. At the end of the experiment (T15), the separation between the oil slick and the medium was still observed in NA, while for BS the blending between the petroleum and the medium was starting.

**Figure 4:**
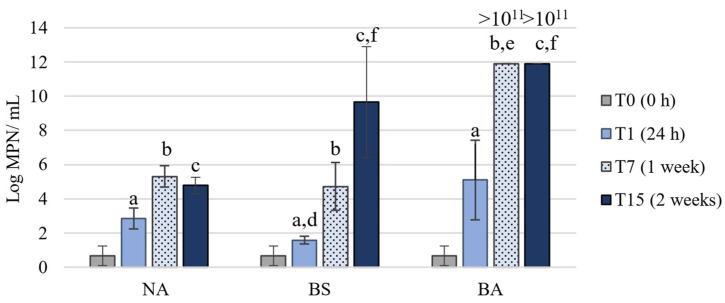
Abundance of hydrocarbons degraders, evaluated by the most-probable number (MPN) method, in the microcosm experiments (mean value, standard deviation, *n* = 3) with different treatments: Natural attenuation (NA), biostimulation (BS) and bioaugmentation (BA). a—significant differences between T1 and T0, for the same treatment; b—significant differences between T1 and T7, for the same treatment; c—significant differences between T1 and T15, for the same treatment; d—significant differences comparing all treatments with NA at T1; e—significant differences comparing all treatments with NA at T7; f—significant differences comparing all treatments with NA at T15.

Regarding the abundance of hydrocarbon degraders (Figure 4), the bioaugmentation (BA) treatment presented, as expected, high values after 24 h of the experiment (10^5^ MPN/mL), significantly higher than values in the BS treatment at the same time (in the order of 10^1^ MPN/mL). Those high levels were only achieved in the NA and BS treatments after 7 days of the experiment. From T7 to T15, the NA treatment maintained the hydrocarbon-degraders abundance, whereas the BS treatment significantly increased the abundance of hydrocarbon degraders, reaching values close to 10^10^ MPN/mL. At the end of the experiment (T15), no significant differences in terms of abundance of hydrocarbon degraders were observed between the treatments BS and BA. However, for BA treatment the values of hydrocarbon degraders were already higher than 10^11^ MPN/mL at T7, showing the tremendous potential of the bacterial consortium for the degradation of hydrocarbons.

**Figure 5:**
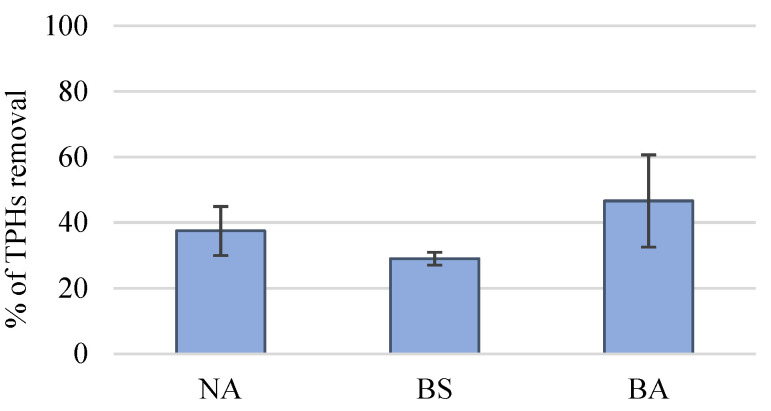
Removal percentage of total petroleum hydrocarbons (TPHs) in the microcosm experiments (mean value, standard deviation, *n* = 3) for the different treatments: natural attenuation (NA), biostimulation (BS) and bioaugmentation (BA).

After 2 weeks of the microcosm experiment (T15), the percentage of TPHs removal was evaluated for each treatment. BA treatment was able to remove the highest percentage of TPHs, 47%, higher than the BS treatment (29%) or NA (37%), although differences were only significant between BA and BS (Figure 5). This result highlights the role of the enriched natural community, after 15 days exposed to petroleum, in the degradation of petroleum hydrocarbons. Probably the petroleum hydrocarbons initially present were gradually degraded in smaller hydrocarbons before complete degradation. Therefore, if the type of petroleum hydrocarbons had been analyzed using a chromatographic methodology, the extend of the different biodegradation levels in the three treatments could have been better shown.

**Figure 6:**
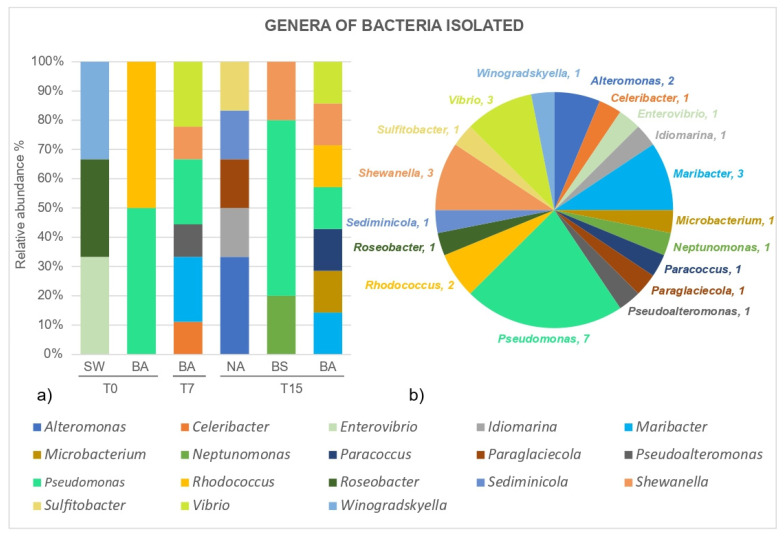
Genera of the bacterial strains obtained in the microcosm bioremediation experiment. (**a**) Relative abundance of the obtained bacterial genera in the natural seawater (SW), in the bioaugmentation (BA) treatment at the beginning (T0), after 7 days of experiment (T7) and at the end of the experiment (after 15 days) (T15), and in the natural attenuation (NA) and biostimulation (BS) treatment after 15 days (T15). (**b**) Total number of bacteria genera obtained in all treatments of the microcosm bioremediation experiment.

Bacterial strains isolated from aliquots of the natural seawater at T0 and of the treatments natural attenuation (NA) and biostimulation (BS) at T15, as well as of the treatment bioaugmentation (BA) at T0, T7 and T15 were identified phylogenetically (Table S1). In a total of 32 bacterial strains isolated and identified throughout the experiment, most of the obtained bacteria belong to the class *Gammaproteobacteria*. Nevertheless, bacteria from the class *Alphaproteobacteria*, *Flavobacteriia* and *Actinobacteria* were also recovered. Overall, 18 different bacterial genera were identified (Figure 6). In the natural seawater (SW) 3 different genera were found. and after a 15 day-exposure to petroleum, it was possible to recover, in the NA treatment, 5 bacterial strains distinct from the ones isolated at the same time in the BS and BA treatment. In BS, only 3 genera were recovered at T15, with a dominance of bacteria belonging to the *Pseudomonas* genus.

For the BA treatment, at the beginning of the experiment (T0) only the two introduced bacterial genera (*Pseudomonas* and *Rhodococcus*) were recovered, maybe due to the high density of the added inoculum used. The relative abundance of the introduced genera *Pseudomonas* and *Rhodococcus*, decreased along time in BA, starting at T0 with 50% each, 0.22% and 0% in T7 and, 0.14% and 0.14% at T15, respectively. After one week (T7), the diversity of the genera recovered from the BA treatment increased. After 15 days (T15) the highest number of genera was recovered in the BA treatment followed by NA.

**Figure 7:**
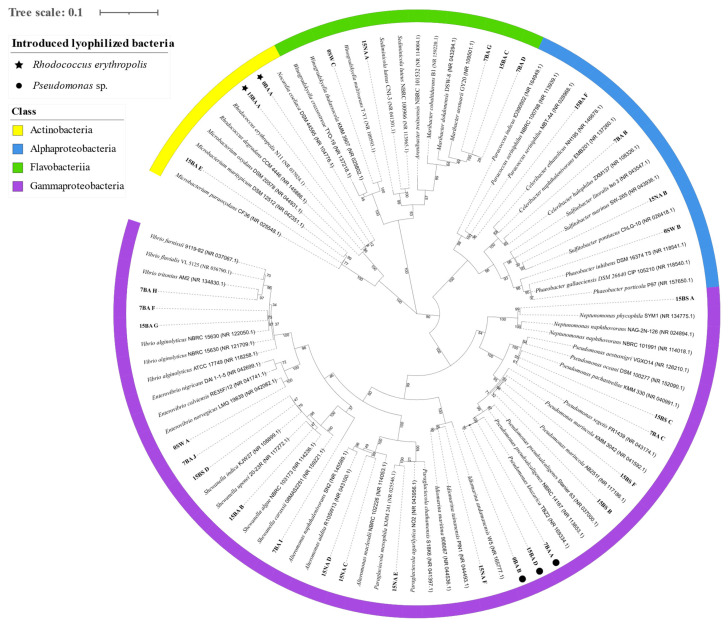
Phylogenetic tree of the bacterial strains isolated in the microcosm bioremediation experiment and their GenBank 3 nearest neighbors. Bacterial isolates were identified in the natural seawater (SW), in the bioaugmentation (BA) treatment at the beginning (T0), after 7 days of experiment (T7) and at the end of the experiment (after 15 days) (T15), and for natural attenuation (NA) and biostimulation (BS) treatment after 15days (T15). The maximum likelihood phylogenetic tree was performed in MEGA X using 93 nucleotide sequences, with 1410 bp length and the bootstrap method with 1000 replications. The tree is drawn to scale, with branch lengths measured in the number of substitutions per site. The GenBank accession numbers are indicated in parenthesis.

The analysis of the constructed phylogenetic tree (Figure 7) shows that both species of the lyophilized bacteria added in the BA treatments were successfully recovered at T0 (0BA_A, 0BA_B) and after 15 days of the experiment (15BA_A, 15BA_D). At T7 only the *Pseudomonas* strain was recovered (7BA_A), not being possible to recover the *Rhodococcus* species. Furthermore, it was possible to recover some other bacterial strains from the different treatments. These bacteria are originated from the natural seawater used in the microcosms experiments that were able to prosper in the presence of petroleum.

## 4. Discussion

It is widely accepted that bioremediation is an eco-friendly, cost-efficient, and effective technique to remediate oil-polluted environments and that the effectiveness of the biodegradation process can be enhanced when complemented with biostimulation and bioaugmentation [41,42]. Moreover, by choosing to apply autochthonous microorganisms in the bioremediation of an oil spill, the degradation performance can be enhanced, as these microorganisms, already adapted to the environment, will be able to compete with the natural community without disclosing the unknown consequences associated to the input of exogenous microorganisms. There are several studies reporting the potential of autochthonous bioaugmentation for bioremediation of hydrocarbons in microcosms [4,20,43,44] and mesocosms experiments [45]. However, the transition from small-scale experiments to larger scale, from microcosms to mesocosms or into the field, can be challenging, due to the complexity of using natural seawater and natural environmental conditions. Another challenge with applying bioaugmentation at a larger bioremediation scale is how to assure the high bacterial biomass input needed and the ratio of biomass/petroleum for an effective bioremediation action.

In the present work, the growth of three oil-degrading bacterial strains (CPN2, CPN3, 1.7 L), belonging to the genera *Rhodococcus* and *Pseudomonas* was optimized, using carbon sources alternative to hydrocarbons (sodium acetate, glucose, glycerol and peptone) for biomass scale-up in bioreactors. Sodium acetate is a rapidly metabolized carbon source, that has proven to enhance biomass growth in bioremediation studies without compromising the degradation ability of the bacterial strains either for hydrocarbons [8] or other organic pollutants [46,47,48]. Glycerol is a simple carbon source, relatively cheap and with increasing interest for the scale up of bacterial biomass production, which has been already used to grow *Rhodococcus* and *Pseudomonas* species [49,50]. Glucose and peptone were also chosen in the preliminary growth experiments as these simple carbon sources are commonly used in the scale-up of bacterial biomass production in biotechnological companies. The *Rhodococcus erythropolis* species CPN2 and CPN3 had higher biomass growth on sodium acetate and glucose, reaching the stationary phase after 48h of incubation, while the *Pseudomonas* sp. (1.7L) grew better in peptone and glucose.

The biomass of these bacterial strains was then scaled-up in bioreactors and lyophilized in the biotechnological company Biotrend. By using the lyophilization technique, the bacterial cells can be preserved for longer periods of time than liquid cultures, also facilitating its storage. Laboratory tests demonstrated that the bacterial strains remained viable after the lyophilization process, without losing their biodegradation potential. In accordance, Li et al. [51] observed that the lyophilization process of a *Bacillus* strain kept the bacterial cells viable and had little effect on crude oil degradation capacity. In the present study, lyophilized strains displayed potential for hydrocarbon degradation either alone or combined in a consortium. In fact, inoculated medium showed a high abundance of hydrocarbon-degraders (>10^7^ MPN/mL) after direct application of the inoculum and after 1 h of incubation in seawater doped with petroleum. This indicates a rapid response of the lyophilized bacterial biomass when in contact with petroleum, both in artificial and natural media. In previous work, the viability and ability of a developed lyophilized microbial degrading formula (composed of two fungal strains and one bacterial strain) to bioremediate oil spills was observed after its application in a polluted beach in China, in an in situ experiment [52]. The previous work supports a possible in situ application of lyophilized microorganisms to remediate oil spills.

In the present work, the consortium of the 3 strains presented values higher than 10^11^ MPN/mL, after 24 h of incubation in seawater with petroleum and nutrients (SPNI). Further experiments allowed optimizing the ratio bacterial strain biomass/petroleum capable of maintaining the efficiency of hydrocarbons degradation, with high values of abundance of oil degraders (10^11^ MPN/mL). Considering the biomass scale-up process for the application of lyophilized bacteria to a real oil spill scenario, a lower ratio of biomass/petroleum would imply fewer costs in the production process and consequently, in the bioremediation process.

The hydrocarbon-degrading performance of the optimized consortium containing the 3 lyophilized oil-degrading bacterial strains, was tested under simulated natural conditions, in microcosm experiments. The bioaugmentation with the lyophilized consortium promoted a significantly higher abundance of hydrocarbon-degraders, after 24 h (ca. 10^5^ MPN/mL), 7 days (>10^11^ MPN/mL) and 15 days (>10^11^ MPN/mL) of incubation with petroleum, when compared to the natural attenuation and biostimulation treatments. Visually, the bioaugmentation flasks were the only ones where emulsion of the oil/water layer occurred, an indication that the introduced bacterial consortium could accelerate the degradation process and have an important role in petroleum degradation as this blending can result in more bioavailable hydrocarbons. To corroborate this result, the introduced lyophilized bacteria (*Rhodococcus erythropolis* and *Pseudomonas* sp.) were recovered throughout the experiment, as evidenced in the phylogenetic tree. Besides being implicated in the degradation of aliphatic and aromatic hydrocarbons species from the genera *Pseudomonas* and *Rhocococcus erythropolis* have been reported to produce biosurfactants [20,53,54,55]. In the beginning, the introduced bacteria might have dominated the microbial community in BA, being the only species recovered (with 50% relative abundance, each). However, both introduced genera decreased their abundance along time, in BA, as other bacterial strains were isolated, as well. After 7 days, the *Pseudomonas* genera represented 22% of the isolated bacteria in BA, while the *Rhodococcus* was not isolated at this time. After 15 days, the genera *Pseudomonas* and *Rhodococcus* represented only 14% of relative abundance, each, in an equal percentage, however, as the other isolated bacteria genera at T15. These results might suggest an adaptation of the microbial community to a stabilization point where both the introduced bacteria and the bacteria selected from the natural seawater had a role in the degradation of petroleum, after 15 days. Probably the petroleum hydrocarbons initially present were degraded by the introduced isolates into smaller hydrocarbons that are suitable carbon sources for several other bacterial strains present in the natural seawater, thus creating the conditions for the observed rise of prokaryotic diversity. To fully understand this dynamic, a microbial community analysis could be performed in the future. By doing this, Shi et al. [56] observed that the introduced bacterial genera, were dominant in the initial phases of a bioaugmentation microcosm experiment for diesel remediation with natural seawater, but its abundance decreased with time, with the increase of other bacterial strains, a similar result to the present work.

In the present work, other bacterial species were also recovered in the microcosm treatments, after 7 and 15 days, linked in previous studies to the degradation of hydrocarbons, such as the *Gammaproteobacteria Vibrio alginolyticus* [57,58], *Pseudoalteromonas* sp. [59], *Alteromonas* sp. [60], *Neptunomonas phycophila* [61] and the *Alphaproteobacteria Sulfitobacter* sp. [13]. The recently described *Flavobacteriia Maribacter thermophilus* [62], was also recovered, and may have potential for hydrocarbon degradation, despite no other study having yet linked this species to hydrocarbon degradation.

The optimum ratio of the bacterial consortium selected in the present work led to the degradation of 47% of TPHs after 15 days of experiment, a significantly higher removal of TPHs compared with BS (29%), and higher (but not significantly) than NA (37%). The evaluation of TPH was chosen as this methodology includes mostly the quantification of saturated hydrocarbons and these hydrocarbons are normally the first type of hydrocarbons to be degraded, which can occur within days (e.g., [39]). This tool can easily allow evaluating the degradation potential. This result highlights the great role that the enriched natural community played in the degradation of petroleum hydrocarbons. However, these results only account for the end of the experiment (after 15 days). To evaluate if the addition of lyophilized bacteria enhanced early on, the degradation of petroleum hydrocarbons, a TPHs removal analysis could have been performed, after 7 days, when the abundance of oil degraders increased significantly in the BA, compared to other treatments. In a previous work [8], the strains CPN2 and CPN3 were tested in a consortium with 3 other strains (two *Pseudomonas* sp. and *Acinetobacter johnsonii*) and together were able to degrade 66% of TPHs in natural seawater. Contrary to this previous study, in the present work, the microcosm flasks were kept closed. The lower oxygen input to the experiment may explain the lower THPs removal since oxygen promotes the action of oxygenases, strong aliphatic hydrocarbons hydrocarbon-degrading enzymes [17,63], and isolation of the facultative anaerobe *Neptunomonas phycophila* [61] species. Nonetheless, the introduction of the lyophilized consortium accelerated the degradation of petroleum, a feature observed in some other studies. For instance, Li et al. [51] observed a degradation of 44.2% of total saturated hydrocarbons after 30 days of applying freeze-dried cells of a *Bacillus* strain to remediate petroleum. The authors refer to the potential for applying this solid inoculum in ex-situ bioremediation techniques. However, few studies have considered so far the application of lyophilized bacterial agents to remediate hydrocarbons in seawater and none has addressed its application in an autochthonous point-of-view as in our current study. This might be due to the challenges of applying free cells in open waters or coastal areas. To cope with that, some researchers propose immobilizing the bacterial agents into carriers to enhance their biodegradation efficiency [28,30,31]. For instance, Junusmin et al. [64] observed that a freeze-dried bacterial consortium (composed of two species of *Bacillus* and one *Enterobacter* species) immobilized onto different carriers could degrade up to 93% TPHs after 28 days incubation with crude oil. Luo et al. [65] reported a great potential for mesocoms field application of a freeze-dried bacterial consortium (composed of three *Acinetobacter* strains and one *Gordonia* species) immobilized onto a carrier, after removing 98% of petroleum from the surface of seawater, in 24 h. So, in the future, this approach could be tested for our autochthonous lyophilized consortium.

To fully understand the applicability range of the lyophilized consortium optimized in the present work for large-scale autochthonous bioremediation, future studies should evaluate the biodegradation efficiency of the consortium when subjected to different ranges of environmental parameters like pH, temperature, and salinity. Furthermore, monitoring the degradation of different hydrocarbon families and evaluating biosurfactants production during the bioremediation experiments could be taken into account.

The use of autochthonous microorganisms in the application of a remediation product might be key to assure the effectiveness of the bioremediation product when considering its application in a real oil-spill scenario. Nevertheless, to tackle the difficulties of applying free-cell bioremediation agents, the application of these bioremediation agents could be complemented with other existing remediation techniques, such as mechanical removal by sorbents and tested in future studies. Commercially available microbial agents for hydrocarbon bioremediation, belonging mostly to the bacteria group, includes species from the genera *Pseudomonas* and *Rhodococcus* [33]. However, there is still a low number of patents for marine oil spill bioremediation and scarce patents for autochthonous bacterial agents [34]. Hence, more research and development in this area is needed.

## 5. Conclusions

In this work, a lyophilized bioremediation agent based on three strains of hydrocarbon-degrading bacteria isolated from the NW Portuguese coast was developed. The lyophilization process did not compromise the bacteria’s ability to degrade hydrocarbons nor cell viability. The consortium of the three lyophilized bacteria proved to enhance the petroleum hydrocarbons degradation performance, when applied as an autochthonous bioaugmentation inoculum to microcosm experiments with natural seawater and nutrients. Moreover, these bacterial strains added to the natural seawater were recovered at the end of the experiment. To the best of our knowledge, this is the first study to develop and optimize the biomass ratio of a lyophilized bioremediation agent based on autochthonous hydrocarbon-degrading bacteria, to remediate petroleum in simulated natural conditions. Given the ubiquity of oil-degrading bacteria, the formulation of tailor-made bioremediation agents, with autochthonous bacteria, can be adapted for other geographical areas, to tackle oil spills.

Future studies should test the efficiency of the developed bioremediation agent in mesocosm and in situ experiments, test the shelf-life of the bacterial agent and test its efficiency to degrade other hydrocarbon contaminants, such as maritime fuels.

## Data Availability

The data presented in this study are available in the current article and the respective Supplementary Materials.

## Supplementary Materials

The following are available online at https://www.mdpi.com/article/10.3390/microorganisms9112285/s1, Figure S1: Biomass growth (mean value, standard deviation, *n* = 3) of the bacterial isolates CPN2, CPN3 and 1.7 L with different carbon sources (10 g·L^−1^), Figure S2: Representative scheme of the experiment with the lyophilized strains CPN2 (2i), CPN3 (3i), 1.7 L (4i) and a consortium (5i), in the controls SP (sterile seawater + petroleum), SPN (sterile seawater + petroleum + nutrients) and the treatments SPI (sterile seawater + petroleum + inoculum), SPNI (sterile seawater + petroleum + nutrients + inoculum), NBPI (nutrient broth + petroleum + inoculum) and BHI (bushnell-haas + inoculum), Figure S3: Representative scheme of the experiment with the controls SP (natural seawater + petroleum), SPN (natural seawater + petroleum + nutrients) and with different concentrations of the consortium of the lyophilized strain: 6i (1.7 g L^−1^), 7i (1.7 × 10^−1^ g L^−1^) and 8i (1.7 × 10^−2^ g L^−1^), applied to the treatments SPI (natural seawater + petroleum + inoculum) and SPNI (natural seawater + petroleum + nutrients + inoculum), Figure S4: Representative scheme of the microcosms experiments with natural seawater (SW), petroleum and the optimum concentration of the consortium of lyophilized strains 7i (1.7 × 10^−1^ g L^−1^), with the treatments natural attenuation, bioaugmentation and bioaugmentation and Table S1: Phylogenetic identification of bacterial strains isolated in the microcosms bioremediation experiment for the natural seawater at T0 (0SW) and for the different treatments: natural attenuation (NA) and biostimulation (BS) at T15, and bioaugmentation (BA) at T0, T7 and T15.
