# Supplementary material for: Bioremediation of Petroleum Hydrocarbons in Seawater: Prospects of Using Lyophilized Native Hydrocarbon-Degrading Bacteria
Source: Microorganisms. 2021 Nov 3;9(11):2285. doi: 10.3390/microorganisms9112285 (PMC8617842; doi:10.3390/microorganisms9112285)
Supplement: Supplementary file 1 [file microorganisms-09-02285-s001.zip › microorganisms-1402365-supplementary.pdf]

Supplementary Materials

Manuscript Perdigão et al.

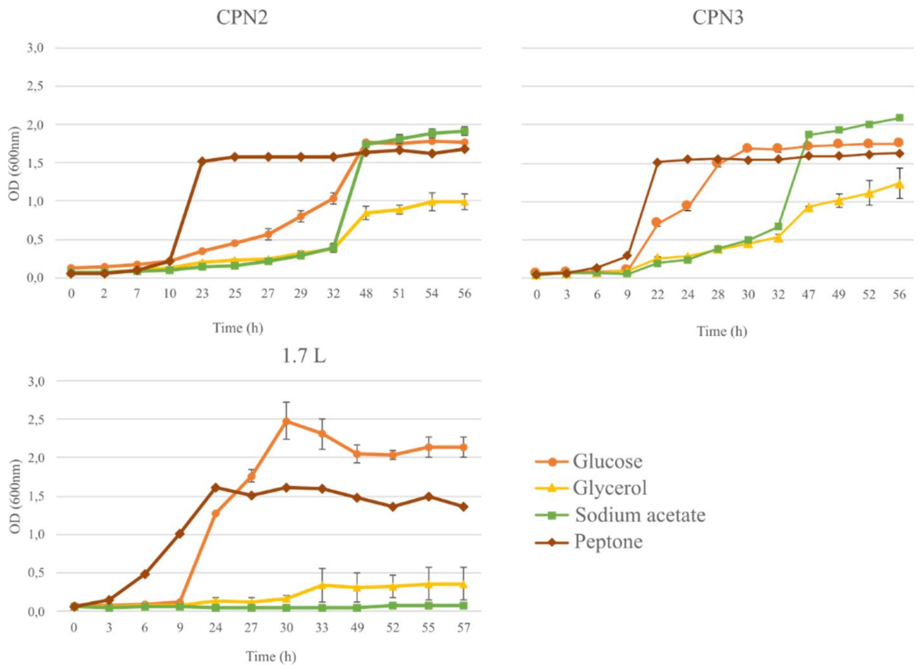

Figure S1. Biomass growth (mean value, standard deviation, n=3) of the bacterial isolates CPN2, CPN3 and 1.7 L with different carbon sources (10 g.L^{-1}).

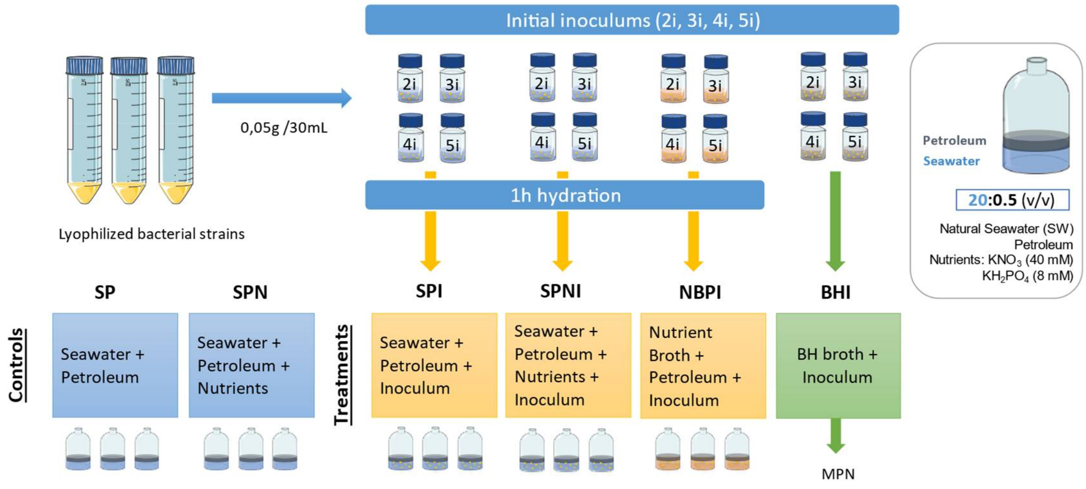

Figure S2. Representative scheme of the experiment with the lyophilized strains CPN2 (2i), CPN3 (3i), 1.7 L (4i) and a consortium (5i), in the controls SP (sterile seawater + petroleum), SPN (sterile seawater + petroleum + nutrients) and the treatments SPI (sterile seawater + petroleum + inoculum), SPNI (sterile seawater + petroleum + nutrients + inoculum), NBPI (nutrient broth + petroleum + inoculum) and BHI (bushnell-haas + inoculum).

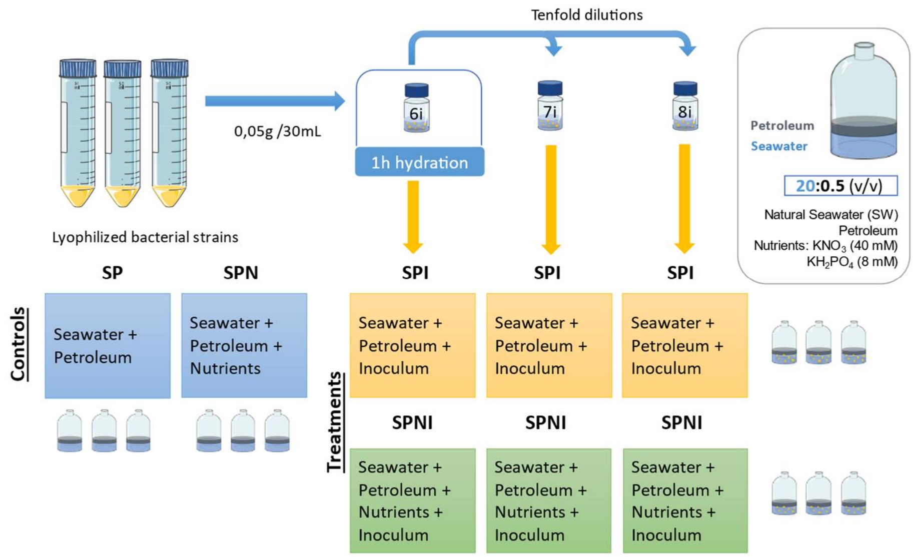

Figure S3. Representative scheme of the experiment with the controls SP (natural seawater + petroleum), SPN (natural seawater + petroleum + nutrients) and with different concentrations of the consortium of the lyophilized strain: 6i (1.7 g L^{-1}), 7i (1.7*10^{-1} g L^{-1}) and 8i (1.7*10^{-2} g L^{-1}), applied to the treatments SPI (natural seawater + petroleum + inoculum) and SPNI (natural seawater + petroleum + nutrients + inoculum).

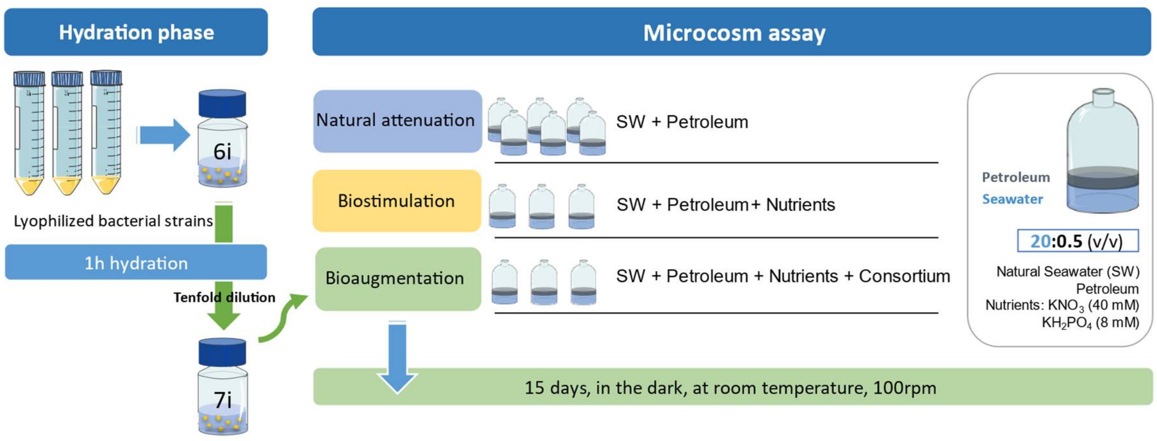

Figure S4. Representative scheme of the microcosms experiments with natural seawater (SW), petroleum and the optimum concentration of the consortium of lyophilized strains 7i (1.7*10^{-1} g L^{-1}), with the treatments natural attenuation, bioaugmentation and bioaugmentation.

Table S1. Phylogenetic identification of bacterial strains isolated in the microcosms bioremediation experiment for the natural seawater at T0 (0SW) and for the different treatments: natural attenuation (NA) and biostimulation (BS) at T15, and bioaugmentation (BA) at T0, T7 and T15.

Isolates	Closest Identification	Sequence length	% similarity*	Class	Accession number
1.7 L	*Pseudomonas sp.*	1407	99.86	Gammaproteobacteria	OK482776
0SW_A	*Enterovibrio* sp.	1392	99.64	Gammaproteobacteria	MW486132
0SW_B	Rhodobacteraceae**	1333	99.63	Alphaproteobacteria	MW486149
0SW_C	*Winogradskyella multivorans*	1365	99.71	Flavobacteriia	MW486161
**0BA_A**[a]	*Rhodococcus erythropolis*	1386	99.93	Actinobacteria	MW486151
**0BA_B**[a]	*Pseudomonas* sp.	1408	99.86	Gammaproteobacteria	MW486147
7BA_A	*Pseudomonas oleovorans*	1409	99.79	Gammaproteobacteria	MW486144
7BA_B	Rhodobacteraceae**	1335	99.10	Alphaproteobacteria	MW486150
7BA_C	*Pseudomonas pachastrellae*	1412	99.86	Gammaproteobacteria	MW486146
7BA_D	*Maribacter thermophilus*	1401	99.29	Flavobacteriia	MW486135
7BA_F	*Vibrio alginolyticus*	1426	99.86	Gammaproteobacteria	MW486158
7BA_G	*Maribacter thermophilus*	1404	99.07	Flavobacteriia	MW486136
7BA_H	*Vibrio tritonius*	1374	99.71	Gammaproteobacteria	MW486160
7BA_I	*Pseudoalteromonas* sp.	1404	99.93	Gammaproteobacteria	MW486141
7BA_J	*Shewanella* sp.	1415	99.72	Gammaproteobacteria	MW486156
15NA_A	*Sediminicola luteus*	1389	98.78	Flavobacteriia	MW486153
15NA_B	*Sulfitobacter pontiacus*	1335	99.78	Alphaproteobacteria	MW486157
15NA_C	*Alteromonas* sp.	1408	99.57	Gammaproteobacteria	MW486130
15NA_D	*Alteromonas* sp.	1404	99.64	Gammaproteobacteria	MW486131
15NA_E	*Paraglaciecola mesophila.*	1401	99.57	Gammaproteobacteria	MW486140
15NA_F	*Idiomarina* sp.	1416	99.79	Gammaproteobacteria	MW486133
15BS_A	*Neptunomonas phycophila*	1410	99.72	Gammaproteobacteria	MW486138
15BS_B	*Pseudomonas marincola*	1399	99.86	Gammaproteobacteria	MW486142
15BS_C	*Pseudomonas pachastrellae*	1410	99.79	Gammaproteobacteria	MW486145
15BS_D	*Shewanella* sp.	1417	99.58	Gammaproteobacteria	MW486155
15BS_F	*Pseudomonas marincola*	1413	99.79	Gammaproteobacteria	MW486143
**15BA_A**[a]	*Rhodococcus erythropolis*	1391	99,86	Actinobacteria	MW486152
15BA_B	*Shewanella* sp.	1418	99,58	Gammaproteobacteria	MW486154
15BA_C	*Maribacter thermophilus*	1405	98,93	Flavobacteriia	MW486134
**15BA_D**[a]	*Pseudomonas oleovorans*	1406	99,79	Gammaproteobacteria	MW486144
15BA_E	*Microbacterium paraoxydans*	1391	99,93	Actinobacteria	MW486137
15BA_F	*Paracoccus seriniphilus*	1337	99,63	Alphaproteobacteria	MW486139
15BA_G	*Vibrio* sp.	1402	99,86	Gammaproteobacteria	MW486159

* similarity percentages according to the nucleotide collection database of the NCBI; ** the identification of the isolates was made only until the family level; [a] isolate similar to the introduced lyophilized bacterial strains (CPN2, CPN3 and 1.7L) used as inocula for the bioaugmentation treatments.
